# Impact of cochlear implants use on voice production and quality

**DOI:** 10.1038/s41598-024-63688-3

**Published:** 2024-06-04

**Authors:** Angela Guastamacchia, Andrea Albera, Giuseppina Emma Puglisi, Charles J. Nudelman, Simin Soleimanifar, Arianna Astolfi, Justin M. Aronoff, Pasquale Bottalico

**Affiliations:** 1 Department of Energy, Politecnico di Torino, 10129 Turin, Italy; 2 Department of Surgical Sciences, Universitá degli Studi di Torino, 10100 Turin, Italy; 3 Department of Speech and Hearing Science, University of Illinois Urbana-Champaign, Champaign, IL 61820 USA

**Keywords:** Voice quality, Cochlear implants, Hearing loss, Acoustic voice quality index, Health care, Medical research

## Abstract

Cochlear implant users experience difficulties controlling their vocalizations compared to normal hearing peers. However, less is known about their voice quality. The primary aim of the present study was to determine if cochlear implant users’ voice quality would be categorized as dysphonic by the Acoustic Voice Quality Index (AVQI) and smoothed cepstral peak prominence (CPPS). A secondary aim was to determine if vocal quality is further impacted when using bilateral implants compared to using only one implant. The final aim was to determine how residual hearing impacts voice quality. Twenty-seven cochlear implant users participated in the present study and were recorded while sustaining a vowel and while reading a standardized passage. These recordings were analyzed to calculate the AVQI and CPPS. The results indicate that CI users’ voice quality was detrimentally affected by using their CI, raising to the level of a dysphonic voice. Specifically, when using their CI, mean AVQI scores were 4.0 and mean CPPS values were 11.4 dB, which indicates dysphonia. There were no significant differences in voice quality when comparing participants with bilateral implants to those with one implant. Finally, for participants with residual hearing, as hearing thresholds worsened, the likelihood of a dysphonic voice decreased.

## Introduction

Studies indicate that although Cochlear Implants (CIs) might not completely restore hearing levels^1,2^, they can aid in enhancing speech comprehension in quiet^3,4^. However, the impact of CIs on various aspects of vocal production in CI users remains unclear. For instance, although there is evidence that access to auditory feedback through CIs could improve vocal pitch control^5,6^, such effects are not consistent across studies (e.g.,^7,8^). To date, it has been difficult to predict a consistent pattern of vocal characteristics for CI users (e.g.,^7,9,10^), and the prevalence of voice disorders within CI users is unknown. The voice quality of individuals with hearing impairment has been broadly described as strained and breathy (e.g.,^11^). These impairments in perceptual voice quality symptoms are speculated to be caused by imbalances between respiratory-laryngeal function (i.e., uncoordinated vocal technique)^12,13^.

Voice quality is a multidimensional construct that is not robustly quantified by unidimensional measures, such as fundamental frequency (i.e., measured in Hz) or intensity (i.e., measured in dB)^14^. Rather, voice quality is best reflected by the harmonic structure of the voice signal, something poorly encoded by CIs. Two factors negatively affect the encoding of harmonic structures by CIs. First, CIs encode the envelopes for broad frequency regions, breaking the incoming acoustic signal into a small number of “channels”. The CI does not encode how many harmonics fall within a channel, and if multiple harmonics fall within one channel, they will be combined, making them unresolvable. Second, stimulation does not encode where within the frequency region encoded by a channel the harmonic occurred, meaning that the spectral relationship between harmonics encoded by different channels is likely distorted.

Given the importance of harmonic structure for voice quality, measures within the frequency domain and quefrency domain are typically adopted in the diagnosis of impaired voice quality associated with dysphonia^15^. One well-known multivariate voice quality metric that captures the harmonic structure of a voice signal is the Acoustic Voice Quality Index (AVQI)^16^. The AVQI provides an ecologically valid (i.e., representative of daily voice use) index of dysphonia severity^17^. As an index, it ranges from 0 (normal) to 10 (severe dysphonia). Generally, scores below 2.50 on the AVQI are considered normal, scores between 2.50 and 4.17 are considered mild dysphonia, scores between 4.17 and 6.23 are considered moderate dysphonia, and scores above 6.23 are considered severe dysphonia^18^. A recent study^19^ confirmed the AVQI as a valid tool for the acoustic measurement of overall voice quality for the Italian speaking population. The authors found a similar threshold (2.35) to discriminate between normal and hoarse voices. To differentiate between mild and moderate dysphonia, the thresholds proposed by Shabnam et al.^18^ have been used. To derive the AVQI, a weighted combination of six acoustic parameters (smoothed cepstral peak prominence (CPPs); harmonic-to-noise ratio (HNR); shimmer local and dB (Shim, ShdB); the general slope of the spectrum (Slope) and tilt of the regression line through the spectrum (Tilt)) is modeled in a linear regression formula.

Outside of a combined index, CPPS as a single measure is widely adopted in the diagnosis of voice disorders as part of a multidimensional assessment^20–22^ Measures of CPPS provide the magnitude of the cepstral peak relative to the amplitude of phonation^23^. This peak reflects the degree of harmonicity in the voice signal and can demonstrate the extent to which the voice signal separates from “noise”^24^. The CPPS cutoff value is 11.46 dB^25^ which means that values lower than 11.46 dB reflect dysphonia, as there is significantly less of a degree of separation of the voice signal from noise. Of note, this cutoff value will vary depending on the software used. The first goal of this study is to determine if CI users have dysphonic voices, as measured by AVQI and CPPS, when using their CIs.

One additional concern regarding CI users’ voices is that bilateral CI use can detrimentally affect voice control compared to unilateral CI use^8,26^. This could stem from incongruent acoustic feedback between the ears^27–29^ or from the fusion of various pitches into one auditory perception when both ears are engaged^30^, which lowers spectral resolution and may further degrade the encoded harmonic structure in the auditory feedback. As such, the second goal of this study is to determine if bilateral CI users’ voice quality is worse than that of unilateral CI users. A final consideration regarding CI users’ voices is that residual hearing in bimodal CI users (i.e., using one hearing aid, HA, and one CI) could detrimentally affect voice quality. This occurs due to the mismatch between the acoustic hearing ear (HA) and the CI ear. In other words, voice quality is hypothesized to be reduced when combining the perceptually distinct acoustic input (from one HA) and the CI input. Ostensibly, this reduction in voice quality would occur in bimodal users for similar reasons to those previously mentioned (i.e., acoustic feedback between the ears is markedly different which may further degrade the encoded harmonic structure in the auditory feedback). Thus, the third goal of this study is to determine how the presence of residual hearing in bimodal (HA-CI) users impacts voice quality.

## Results

Analyses were stratified according to the hearing modalities within our sample, resulting in three distinct analyses:

1. A within-participants analysis that compared the effects of having the CI device turned on versus off, on voice parameters, independent of the participant’s status as a unilateral or bilateral CI user (n = 20), using LME models;
2. A between-participants (9 bilateral vs. 11 unilateral CI users) comparison which analyzed the variation ($\Delta$) in the voice parameters in the on and off conditions, using independent samples t-tests;
3. A within-participant analysis that assessed only bimodal users (CI and hearing aid) when both the devices were on (n = 7). In all the models, sex is present as an independent variable only when statistically significant.

### Within participants analysis

**Figure 1:**
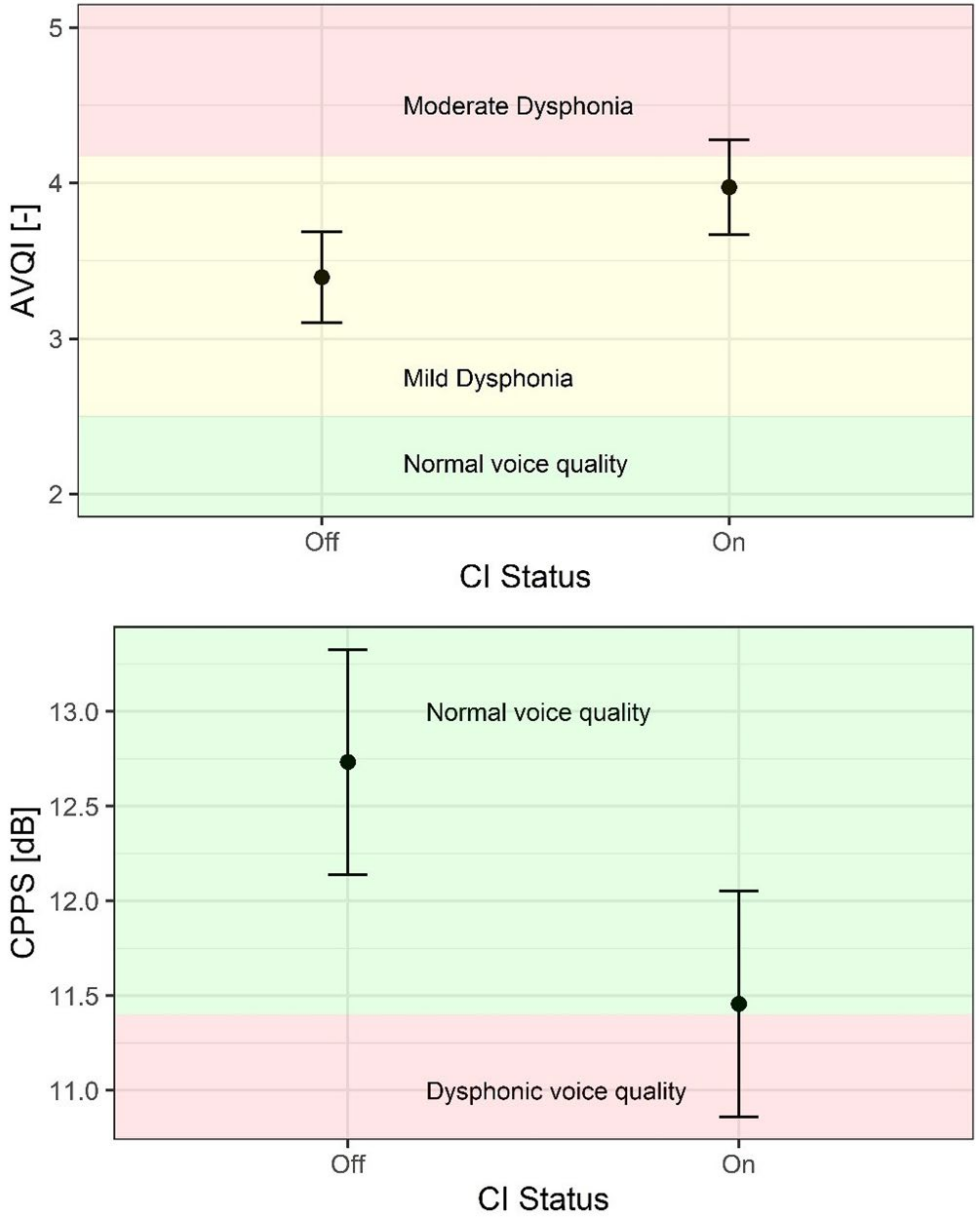
Mean values and the standard errors in the two CI conditions (on and off) for AVQI, and CPPS.

A within-participants analysis that compared the effects of having the CI device turned on versus off, on voice parameters, independent of the participant’s status as a unilateral or bilateral CI user, was completed using LME models. The mean values and the standard errors for the two different voice parameters are shown in Fig. 1, while the estimate, standard error, degrees of freedom, *t*-value, and *p*-value for the fixed factor (CI on vs. CI off) are displayed in Table 1. For the AVQI model, the conditional $R^2$, interpreted as a variance explained by the entire model, including both fixed and random effects, was 0.81. According to Cohen^31^, an $R^2$ for a multiple regression model equal to or greater than 0.26 is considered to be a strong effect size. No effects of sex or age were detected. Participant ID was used as a random factor, removing 1.38 of the variance from the model and leaving a residual variance of 0.39. Participant ID removed more variance than the residual variance. This suggests that the inclusion of participant ID as a random factor effectively reduces a significant amount of variability in the AVQI scores. The implication is that the random factor is an appropriate choice for capturing and accounting for individual differences between participants, thereby improving the model’s validity and reliability. The AVQI was calculated by combining vowel and connected speech stimuli. AVQI values were significantly higher when the CI system was on (*p* = 0.009). Mean AVQI score was 3.4 when the CI system was off, and 4.0 when the CI system was on. Of note, for normal hearing subjects, AVQI scores below 2.50 are considered normal, scores between 2.50 and 4.17 are considered mild dysphonia, scores between 4.17 and 6.23 are considered moderate dysphonia, and scores above 6.23 are considered severe dysphonia^18^. The statistical power of this model was 85%.

For the CPPS model, participant ID was used as a random factor, removing 5.77 of the variance from the model and leaving a residual variance of 1.27. Also in this case, participant ID removed more variance than the residual variance. The conditional $R^2$ was 0.84, showing a strong effect size. The mean CPPS values calculated in the speech tasks were significantly lower when the CI system was on (*p* = 0.003). The mean CPPS was 12.7 dB when the CI system was off and 11.4 dB when the CI system was on. Of note, for normal hearing subjects, CPPS values lower than 11.5 dB reflect dysphonia^25^. The statistical power of this model was 95%.

**Table 1:** LME models output run with AVQI and CPPS as response variables and cochlear implant status (on vs. off), age, and sex as the fixed factors.

Fixed factors	Estimate (–)	Std. Error (–)	df	*t*-value	*p*-value
AVQI					
(Intercept)	3.39	0.30	23.7	11.38	$$<0.001^{***}$$
CI on	0.58	0.20	19.0	2.91	$$0.009^{**}$$
Age	0.01	0.02	17.0	0.23	0.822
Sex male	0.36	0.62	17.0	0.58	0.57
CPPS					
(Intercept)	12.73	0.59	22.7	21.45	$$<0.001^{***}$$
CI on	− 1.28	0.36	19.0	− 3.57	$$0.003^{**}$$
Age	0.02	0.05	17.0	0.35	0.727
Sex male	0.47	1.25	17.0	0.37	0.713

Participant ID was used as a random factor. Significance codes for the *p*-values: *** < 0.001, ** < 0.01.

### Between participants analysis

Regarding the bilateral CI users, the mean AVQI when the CI was off was approximately 3.35 (± 0.35), while when the CI was on, it increased to approximately 3.98 (± 0.46). Regarding the unilateral CI users, the mean AVQI when the CI was off was approximately 3.43 (± 0.46), while when the CI was on, it increased to approximately 3.97 (± 0.42). For AVQI, the difference between the on and off conditions ($\Delta$) was 0.63 and 0.54 for bilateral and unilateral users, respectively.

Regarding the bilateral CI users, the mean CPPS when the CI was off was approximately 12.24 (± 0.82), while when the CI was on, it decreased to approximately 11.19 (± 0.82). Regarding the unilateral CI users, the mean CPPS when the CI was off was approximately 13.13 (± 0.86), while when the CI was on, it decreased to approximately 11.67 (± 0.88). For CPPS, the difference between the on and off conditions ($\Delta$) was − 1.05 and − 1.46 for bilateral and unilateral users, respectively.

A between-participants (bilateral vs. unilateral CI users) comparison analyzed the variation ($\Delta$) in the two voice parameters in the on and off conditions, using independent samples t-tests. The Shapiro-Wilk normality test conducted on the two variables AVQI and CPPS yielded a test statistic (W) of 0.98 (*p*-value 0.95) and 0.97 (*p*-value 0.78). This indicates that, at the 0.05 significance level, there is no significant evidence to reject the null hypothesis of normality. Hence, the data is reasonably normally distributed.

The estimate, standard error, degrees of freedom, *t*-value, *p*-value for the fixed factor (CI on vs. CI off), and Cohen’s d effect sizes are displayed in Table 2. The degrees of freedom were calculated using the Satterthaite-Welch adjustment^32^. The variation in the voice parameters comparing the two CI statuses (on vs. off) did not differ significantly between the two groups (Bilateral and Unilateral CI users). No effects of sex or age were detected.

**Table 2:** T-tests output to compare the means of two groups (Unilateral vs. Bilateral Ci users) on the variations in the two voice parameters (AVQI and CPPS) obtained in the two CI statuses (on vs. off).

Fixed factors	Mean $\Delta$ Unilateral	Mean $\Delta$ Bilateral	*t*-value	df	*p*-value	Cohen’s d effect size
AVQI	0.54	0.63	− 0.23	14.39	0.823	− 0.11 (negligible)
CPPS	− 1.46	− 1.05	− 0.56	17.04	0.581	− 0.25 (small)

#### Within participants analysis: bimodal users (CI and HA)

An analysis was performed on bimodal users CI/HA when both devices were on, relating the two voice parameters to the pure tone average (PTA) hearing sensitivity. Linear models were used for the bimodal analysis, as there were no repeated measures. The estimate, standard error, degrees of freedom, *t*-value, and *p*-value for each fixed factor are displayed in Table 3.

**Figure 2:**
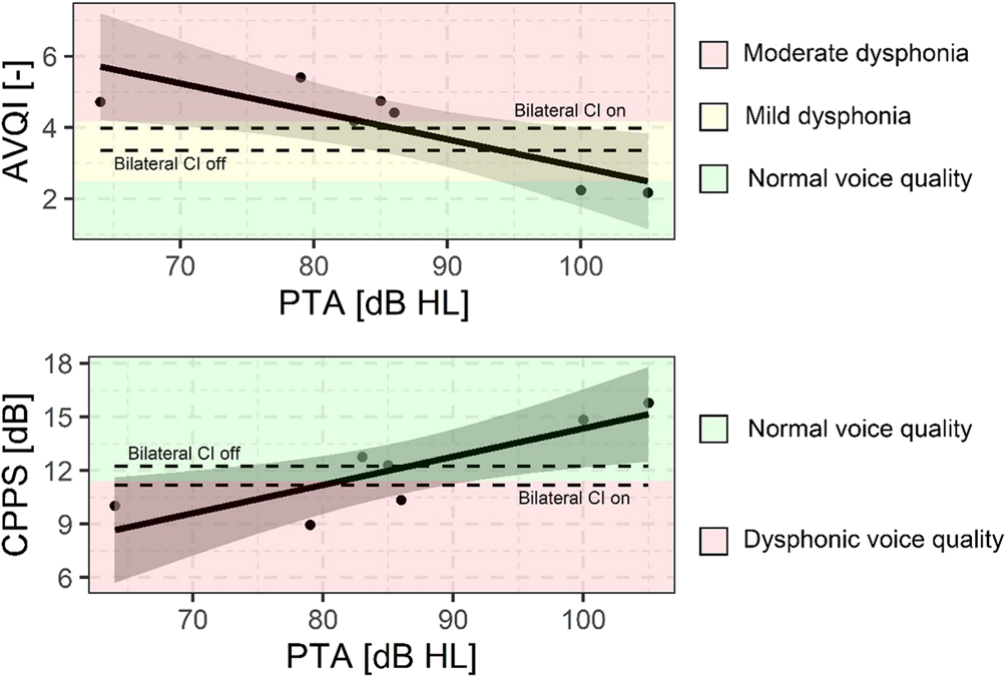
Regression line of AVQI (–) and CPPS (dB) in bimodal CI/hearing aids users with both devices on, versus the residual pure tone average (PTA) sensitivity. As a reference, the mean values of the Bilateral CI users in the two conditions on and off are reported as dashed lines.

The mean AVQI values calculated in the speech tasks were significantly lower (less dysphonic) as PTA thresholds increased (*p* = 0.025). This relationship is displayed in Fig. 2. Mean values of AVQI decreased by 0.08 points for each dB HL increase in PTA threshold. The R-squared of this model was 0.70, while its statistical power was 89%.

The mean CPPS values calculated in the speech tasks were significantly higher (less dysphonic) as PTA thresholds increased (*p* = 0.025). This relationship is displayed in Fig. 2. Mean values of CPPS increased by 0.16 dB for each dB HL increase in PTA threshold. The R-squared of this model was 0.71, while its statistical power was 90%.

**Table 3:** Linear models output run with CPPS, and AVQI as a response variable and Pure Tone Audiometry threshold as the fixed factor.

Fixed factors	Estimate (–)	Std. Error (–)	*t*-value	*p*-value
AVQI				
(Intercept)	13.74	2.36	5.21	$0.013^{*}$
PTA	− 0.09	0.02	− 4.55	$0.019^{*}$
Age	− 0.03	0.02	− 1.45	0.243
Sex male	0.67	0.49	1.36	0.265
CPPS				
(Intercept)	− 1.46	3.94	− 0.37	0.726
PTA	0.17	0.05	3.49	$0.017^{*}$
Age	0.04	0.06	0.68	0.544
Sex male	0.04	1.41	0.03	0.979

Significance codes for the *p*-values: * < 0.05.

## Discussion

The results of the present study indicate that CI users’ voices were dysphonic , and their voice quality was significantly degraded when using their CI. These CI users’ mean AVQI score was 4.0 and mean CPPS value was 11.4 dB. Within the scientific literature, these values indicate a mild-moderate dysphonia^25^. This suggests that the auditory feedback from the CI is not only insufficient for maintaining voice quality but has a detrimental effect. This may reflect the degradation of spectral information that occurs as part of speech processing by the CI, and particularly, the distortion of harmonic structure, as described in the introduction. While previous research has demonstrated that CI users have deficits in terms of CPPS and jitter^10,33^ compared to normal hearing speakers, the current results go beyond that to suggest that the use of the CI itself degrades these voice characteristics.

The results appear to differ from those of Kishon-Rabin et al.^34^ who asked speech therapists to judge the vocal productions of CI users both before and after implantation. They found that the productions of those post-implantation, especially after two years of device use, were rated as better than the pre-implantation (and thus vocalizations with the CI off) productions. Some caution is needed in comparing those results to the current study since the ratings did not separate speech and voice quality and they did not directly compare productions with the CI on and off at comparable time points. It is not clear if the apparent difference with the current study reflects a true difference in results, differences in the conditions being compared, or if CI user’s voice quality without the device improves more post-implantation than their voice when using the CI.

The detrimental effect of using CIs on voice quality is different than what has been seen for fundamental frequency and intensity control. Kirchner et al.^8^ measured bilateral CI users’ ability to maintain a stable fundamental frequency when producing a sustained vowel while either using or not using their CIs. Unlike with voice quality, they did not find a significant shift in fundamental frequency control when the CI was on versus off. This may, in part, reflect the inconsistent benefits seen when using CIs in terms of fundamental frequency control in other studies^5,7^. Previous studies have also found that using a CI improved intensity variability, at least for a subset of participants^5^. The differences in the effects of CI use on fundamental frequency and intensity control versus those on voice quality may reflect the critical role of spectral information for maintaining voice quality, whereas other cues such as amplitude modulation rates and stimulation level can be informative for accurately perceiving fundamental frequency and intensity, respectively^35,36^.

The effects of bilateral versus unilateral CI use differed noticeably from what has been seen for other studies. For example, Kirchner et al.^8^ found that CI users’ fundamental frequency control was significantly worse when using both CIs together compared to their better ear alone. Similarly, Aronoff et al.^26^ found that CI user’s pitch contours when singing “Happy Birthday” were significantly less accurate when using both ears together than when using their better ear alone. The difference between those results and the results from the current study may reflect the different measures used (fundamental frequency versus voice quality). Alternatively, it may reflect the different study design. While both Kirchner et al.^8^ and Aronoff et al.^26^ used a within-subject design and compared performance with both CIs to performance with the better ear alone, the current study directly compared bilateral and unilateral CI users in their usual listening configuration. The within-subject design previously used when investigating fundamental frequency has the advantage of reducing the effects of the commonly seen high variability across CI users, but it also means that in the unilateral condition, participants are using a configuration that they did not typically use in their everyday life, potentially altering how they use the auditory feedback of their voice.

For the bimodal users, as hearing thresholds worsened, the likelihood of a dysphonic voice decreased. This is inconsistent with previous work suggesting that degraded availability or use of auditory feedback can cause voice issues^37–39^. However, it suggests that conflicting information between the two ears, resulting from the use of a CI in the presence of audible acoustic feedback, may lead to a more dysphonic voice. While a detrimental effect was not seen when using two cochlear implants compared to one, it is likely that the difference between the input from the two CIs was small compared to the difference between acoustic input and electric input.

The results from this study indicate that CI users’ voice quality was detrimentally affected by using their CI, raising to the level of a dysphonic voice. This suggests a critical need to investigate the underlying properties of CI processing that lead to this and the design of processing strategies that can restore normal voice quality. Given the present voice acoustic results, it is likely that CI users also experience vocal fatigue^40^, and this highlights the critical need to pursue future research that examines self-reported voice-related quality of life in cochlear implant users^41^.

## Methods

The research was conducted at the University Hospital Cittá della Salute e della Scienza di Torino (Italy) and focused on the assessment of participants’ voice disorder through the evaluation of objective voice parameters. All participants were recruited on a voluntary basis and the project received ethical approval by the Comitato etico interaziendale AOU Cittá della Salute e della Scienza di Torino (IRB # 3546). All methods were carried out in accordance with the relevant guidelines and regulations. The data were transferred to the University of Illinois Urbana-Champaign under the FDP Data Transfer and Use Agreement (ID.109259).

### Participants

After signing an informed consent form, a total of 27 hearing-impaired (HI) patients with at least one cochlear implant (CI) were recruited for the study, including 10 females and 17 males aged 30 to 84 years (mean age = 67.0, Standard deviation = 12.8). All participants were native Italian speakers and had no speech disorders, vocal pathologies, or previous laryngeal surgeries, in order to isolate the effects of hearing deficits and CI usage on voice quality. Additionally, none of the patients were smokers or had worked in noisy environments that required vocal strain. The patients’ hearing impairments had various etiologies, including otosclerosis (3 patients), chronic otitis (5 patients), infective (7 patients), and unknown (12 patients). The study included 9 bilateral CI users, 7 bimodal users (using one hearing aid, HA, and one CI), and 11 unilateral CI users. Only five patients had undergone cochlear implantation surgery less than one year before enrolling in the study. Participants’ characteristics are reported in Table 4.

**Table 4:** Participants’ characteristics (“CI” indicates cochlear implant device and “HA” indicates hearing aid).

ID	Age (years)	Sex	CI status	Cochlear implant device	Pre/post lingual deafness	Device on the left ear (months of use)	Device on the right ear (months of use)
1	48	M	Bimodal	Med-El	Post	HA (21)	CI (8)
2	52	F	Bimodal	Med-El	Post	CI (4)	HA (21)
3	68	M	Unilateral	Ad. Bionics	Post	CI (45)	–
4	55	M	Bilateral	Unknown	Post	CI (202)	CI (106)
5	69	M	Unilateral	Med-El	Post	CI (6)	–
6	78	M	Bimodal	Cochlear	Post	HA (81)	CI (13)
7	74	M	Bilateral	Cochlear	Post	CI (123)	CI (52)
8	60	F	Bilateral	Med-El	Post	CI (167)	CI (28)
9	84	M	Unilateral	Unknown	Post	–	CI (316)
10	74	M	Unilateral	Med-El	Post	CI (202)	–
11	71	M	Unilateral	Ad. Bionics	Post	CI (42)	–
12	79	F	Unilateral	Med-El	Post	CI (70)	–
13	80	M	Unilateral	Med-El	Post	–	CI (155)
14	75	M	Unilateral	Ad. Bionics	Post	CI (48)	–
15	65	M	Unilateral	Cochlear	Post	–	CI (91)
16	73	F	Bimodal	Ad. Bionics	Post	HA (502)	CI (53)
17	69	F	Bilateral	Ad. Bionics	Post	CI (33)	CI (100)
18	70	F	Bilateral	Med-El	Post	CI (273)	CI (29)
19	43	F	Bilateral	Cochlear	Pre	CI (66)	CI (33)
20	79	F	Bimodal	Cochlear	Post	CI (59)	HA (130)
21	70	M	Unilateral	Cochlear	Post	CI (62)	–
22	73	M	Bimodal	Med-El	Post	HA (370)	CI (1)
23	61	M	Bimodal	Med-El	Post	HA (670)	CI (1)
24	55	F	Unilateral	Med-El	Post	CI (157)	–
25	30	M	Bilateral	Med-El	Pre	CI (54)	CI (32)
26	75	M	Bilateral	Cochlear	Post	CI (129)	CI (176)
27	78	F	Bilateral	Unknown	Post	CI (72)	CI (53)

#### Experimental procedure

For each participant, a voice recording was taken while they spoke at a comfortable loudness and pitch, first performing a specific sustained vocalization followed by a text reading. The sustained vocalization consisted of three sustained vowels (/a/, /e/, /i/) lasting for about three seconds. The text used for the reading was a standardized, phonetically balanced passage of 124 words that included a wide range of Italian-language sounds, commonly used for speech recognition testing and articulation drills^20^.

All recordings were taken in quiet conditions inside a partially sound-treated clinic room of the University Hospital Cittá della Salute e della Scienza di Torino. The room was characterized in terms of background noise level and reverberation time. The former was measured in room-occupied conditions for a continuous time interval of four hours employing a calibrated class-1 sound level meter (SLM, model XL2 by NTi Audio, Schaan, Liechtenstein), positioned at a height of 1.2 m from the ground. The background noise level was evaluated as an A-weighted 90th-percentile sound pressure level (LA90), which is the statistical level surpassed for 90% of the measuring time. The reverberation time, in terms of T20, was measured through an impulsive signal generated by Impulsive Sound Source (Model BAS006, Larson Davis, Depew, NY, USA), and acquired at the SLM positioned randomly in the room. T20 measurements were averaged in space and frequency between 250 and 2000 Hz according to the ISO 3382-1 (ISO, 2009). Overall, the sound-treated clinic room was characterized by an LA90 equal to 36 dBA, and a mid-frequency T20 equal to 0.19 s (± 0.01 s).

The microphone used for the voice acquisition was a cardioid condenser microphone AKG Perception 120 (P120, AKG, Vienna, Austria) with a frequency response range of 20–20 kHz, a sensitivity of 24 mV/Pa at 1 kHz, and a maximum output impedance of 200 ohms. The embedded bass-cut filter and the pre-attenuation mechanism were disabled during the recordings. The P120 microphone was chosen based on its performance in a previous study^22^ that compared its performance to that of four other microphones. The data logger used was a four-channel handy recorder ZOOM H4nPro (Zoom Corp., Tokyo, Japan) that acquired the P120 signal in an uncompressed format (.wav) at a sampling frequency of 44.1 kHz and 16-bit resolution.

During the recording, the participant was sitting on a chair facing the table, keeping a mouth-microphone distance of about 25 cm. All tests were performed in quiet conditions keeping all doors and windows closed. For each participant, the speech recordings were performed under two different conditions: one with their devices on (i.e., 2 CIs on for bilateral CI users, 1 CI on for the unilateral CI users, and 1 CI and 1 HA on for the bimodal users), and one with unaided listening (i.e., without the support of any hearing device). The recording lasted about 10 minutes, and for each participant, the order of the recording conditions was randomized.

#### Voice parameters and statistical analysis

The voice samples were analyzed using AVQI and CPPS. All the parameters were calculated with PRAAT version 6.0.13^42^.

The Acoustic Voice Quality Index (AVQI) is a multivariable model based on acoustic measures that permits the objective assessment of overall dysphonia severity using sustained vowel and continuous speech^15^. The AVQI provides an ecologically valid (i.e., representative of daily voice use) index of dysphonia severity^15,17^. It ranges between 0 (normal) and 10 (severe dysphonia). To derive the AVQI, a weighted combination of parameters is modeled in a linear regression formula. The parameters consist of six acoustic measures as follows: time domain (shimmer local, shimmer local dB and harmonics-to-noise ratio), frequency domain (general slope of the spectrum and tilt of the regression line through the spectrum), and quefrency domain (smoothed cepstral peak prominences).

Measures of smoothed cepstral peak prominence (CPPS ) are reliable and strong measures of dysphonia^20–22^, as CPPS is somewhat robust to small errors in fundamental frequency tracking. The CPPS allows for comparison between two testing situations, as it provides the magnitude of the cepstral peak relative to the amplitude of phonation^23^.

Statistical analysis was conducted using R version 4.0.2. Three types of analysis were performed: Linear Mixed-Effects (LME) models, an independent samples t-test, and linear model (LM). Linear Mixed-Effects (LME) models were fit by restricted maximum likelihood (REML), when repeated measurements on the same participant were performed. Random effects terms were chosen based on the variance explained. The LME output includes the estimates of the fixed effects coefficients, the standard error associated with the estimate, the degrees of freedom (df), the test statistic (*t*), and the *p*-value. The Satterthwaite method^32^ is used to approximate degrees of freedom and calculate *p*-values. The LME models were selected, as linear models are statistical tools used to explore and test relationships between variables, when only one measurement was available for each participant. The model assumes that the dependent variable can be expressed as a linear function of one or more independent variables, along with an error term.

The *t*-test was selected as a statistical analysis in order to compare the means of two groups of data. The *t*-test output includes the test statistic (*t*), the degrees of freedom, df, and the *p*-value.

## Data Availability

The datasets presented in this article are not readily available because the database consists of speech recordings, which are considered identifiable data. Requests to access the datasets should be directed to Pasquale Bottalico, pb81@illinois.edu.
